# Specificity of Penicillin Acylases in Deprotection of N-Benzyloxycarbonyl Derivatives of Amino Acids

**DOI:** 10.32607/actanaturae.13703

**Published:** 2023

**Authors:** I. A. Morozova, D. T. Guranda, N. V. Panin, V. K. Švedas

**Affiliations:** Lomonosov Moscow State University, Belozersky Institute of Physicochemical Biology, Moscow, 119234 Russian Federation; Lomonosov Moscow State University, Research Computing Center, Moscow, 119234 Russian Federation; Lomonosov Moscow State University, Faculty of Bioengineering and Bioinformatics, Moscow, 119234 Russian Federation

**Keywords:** penicillin acylases, substrate specificity, enzymatic deprotection of functional groups, N-benzyloxycarbonyl derivatives of amino acids

## Abstract

Changes in the structure of the N-acyl group in N-acylated amino acid derivatives significantly affect both the recognition and activity of penicillin acylases on this series of substrates. However, penicillin acylases from both Alcaligenes faecalis and Escherichia coli are capable of removing the N-benzyloxycarbonyl protecting group in amino acid derivatives under mild conditions without the use of toxic reagents. Efficiency in using penicillin acylases in preparative organic synthesis can be improved by utilizing modern rational enzyme design methods.

## INTRODUCTION

Masking functional groups is an important aspect of organic synthesis [1, 2]. The use of enzymes for introducing and removing protective groups significantly expands the possibilities in this area, up to the application of new reagents and changing the conditions for these stages. Thus, one set of reagents is used in the chemical synthesis of peptides, which is mainly carried out in organic solvents, while, when applying enzymes (e.g., to mask amino groups), one can introduce the phenylacetyl [3], phthalyl [4], and acetyl [5] protective groups. Biocatalysis in organic synthesis, and especially in drug preparation, also aims to search for enzymes that can catalyze traditional chemical reactions and make them more environmentally and economically attractive. One such example is the removal of the benzyloxycarbonyl protecting group in amino compounds, which is conventionally carried out by catalytic hydrogenation, sodium reduction in liquid ammonia, and acidolysis using hydrogen bromide in acetic acid [1, 2]. The following limitations and disadvantages of these methods can be distinguished as follows: the widely used hydrogenation on a palladium catalyst cannot be applied when the structure contains organic sulfides, including cysteine or methionine residues [6]. Deblocking can be carried out in the presence of cyclohexylamine or boron trifluoride etherate [7]. However, the method is not selective in the presence of reducible functional groups such as C=C, C=O, CN, NO_2_, formyl, carbamoyl, etc. [8]. One should also take into account such factors as the toxicity of palladium and the lack of reliable methods for removing its traces from the final product, which is extremely important in drug synthesis [9]. During reductive cleavage with sodium in liquid ammonia [10], other protective groups are simultaneously cleaved off with the benzyloxycarbonyl residue. Ester groups are, at least partially, converted to amides, threonine residues are destroyed, methionine residues are partially demethylated, and some peptide bonds are cleaved. Side reactions of interesterification and acetylation of threonine and serine residues occur during acidolytic cleavage; tryptophan, nitroarginine residues, benzyl esters, and amide groups are destroyed [11]. Along with optimizing the conditions for these reactions in order to reduce the contribution of side processes, employing biocatalytic methods to remove the N-benzyloxycarbonyl protection of amino groups is of interest. New enzymes such as urethane hydrolases were discovered as progress was made in this direction [12, 13]. Enzymes capable of removing benzyloxycarbonyl protection were found in Sphingomonas paucimobilis, Burkholderia phenazinium, and Arthrobacter sp. [14, 15, 16]. The ability of Escherichia coli penicillin acylase to cleave the N-benzyloxycarbonyl derivatives of amino acids was also shown [17]. The aim of this work is to study how changes in the structure of the N-acyl group (replacement of the phenylacetyl residue with benzyloxycarbonyl) alter the specificity of penicillin acylases from Alcaligenes faecalis and Escherichia coli. Furthermore, this study aims to compare the ability of the two enzymes to remove the protective group.

## EXPERIMENTAL

**Reagents**

We used phenylacetyl chloride (Sigma, USA), phenylmethylsulfonyl fluoride (Merck, Germany), and acetonitrile (Cryochrome, Russia) as reagents. The N-phenylacetyl derivatives of α-amino acids were synthesized according to the method described previously [18].

Penicillin acylase from Escherichia coli was prepared using the procedure described earlier [19], while penicillin acylase from Alcaligenes faecalis was procured from LLC Innovations and High Technologies of Moscow State University. The concentration of active sites of penicillin acylases was determined by titration with phenylmethylsulfonyl fluoride (PMSF) as described previously [20, 21].

**Determining the kcat and KM values for penicillin acylase-catalyzed hydrolysis of N-acyl derivatives of amino acids**

Kinetic experiments were carried out in a thermostated cell of a Shimadzu UV-1601 spectrophotometer at 400 nm and 25°C in 0.01 M phosphate buffer pH 7.5 in the presence of 0.1 M KCl. The values of the Michaelis constant K_M_ for the hydrolysis of the N-phenylacetyl and N-benzyloxycarbonyl derivatives of the amino acids were determined as the constants of competitive inhibition of the hydrolysis of the NIPAB chromogenic substrate by these compounds by analyzing the dependence between the observed Michaelis constants of NIPAB hydrolysis on the concentration of the N-phenylacetyl or N-benzyloxycarbonyl derivative of the amino acid. The catalytic constants of enzymatic hydrolysis of the N-phenylacetyl and N-benzyloxycarbonyl amino acid derivatives were determined at saturation with the substrate (concentration numerically equals to 10 K_M_ and 20 K_M_) to determine the maximum rate of the enzymatic reaction.

The progress of the reaction was followed by sampling and spectrophotometric registration of the resulting amino groups after modification with o-phthalaldehyde. In a typical experiment, a solution of the N-acyl amino acid derivative in 0.01 M phosphate buffer (pH 7.5) containing 0.1 M KCl was placed in a thermostated cell at 25°C and the required amount of the enzyme was added under stirring. After some time, samples (15–30 μL) of the reaction mixture were taken, mixed with 50 μL of a 10 mM PMSF solution in isopropanol to stop the reaction, diluted to the desired concentration, and analyzed by HPLC. In order to determine the initial rates of enzymatic hydrolysis, 8–10 samples were typically taken; the substrate conversion did not exceed 10%.

**HPLC analysis with pre-column modification of amino groups with o-phthalaldehyde**

Primary amino groups were modified as follows: 50 µL of a methanol solution containing NAC (40 mM) and o-FA (20 mM) was added to 900 µL of a 0.5 mM solution of an amino compound in 0.4 M borate buffer (pH 9.6) at 25°C. The mixture was stirred, diluted with a chromatography eluent after 15 min, centrifuged for 3 min at 12,000 rpm, and analyzed. The chromatographic system consisted of a Waters M6000 eluent supply module, a Reodyne 7 125 type injector with a 50 µL loop, a Nucleosil C1-8 Chrompack Varian reverse phase chromatography column (250×4 mm, 5 µm), and a Waters M481 LC detector. Chromatograms were recorded using the Multichrome hardware-software system for collecting and processing chromatographic data (Ampersend, Russia). The flow rate was 1 mL/min. The resulting isoindoles were analyzed at 340 nm using 6 mM phosphate buffer (pH 6.8) containing acetonitrile (10–40 vol.%) as the mobile phase.

**Direct HPLC analysis of the reaction mixture components**

HPLC analysis of the reaction mixture components without pre-column modification of the formed amino groups was carried out using a Waters chromatographic system, a Kromasil Eternity-5-C18 column (Eka Chemicals, Sweden), 6 mM phosphate buffer pH 3.0 containing acetonitrile (30 vol.%) and 0.1 g/L so dium dodecyl sulfate at 210 nm, and a flow rate of 1 mL/min.

## RESULTS AND DISCUSSION

**Fig. 1:**
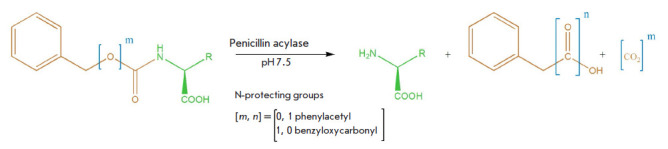
Removal of phenylacetyl and benzyloxycarbonyl protecting groups catalyzed by penicillin acylases

The scheme of enzymatic hydrolysis of the N-phenylacetyl and N-benzyloxycarbonyl derivatives of amino acids is presented in Fig. 1. It is noteworthy that the products of these two reactions differ: when the N-phenylacetyl protection is removed, an amino acid with a free amino group and phenylacetic acid are formed while removal of the N-benzyloxycarbonyl protection leads to the accumulation of benzyl alcohol, along with the amino acid and the release of CO_2_.

**Fig. 2:**
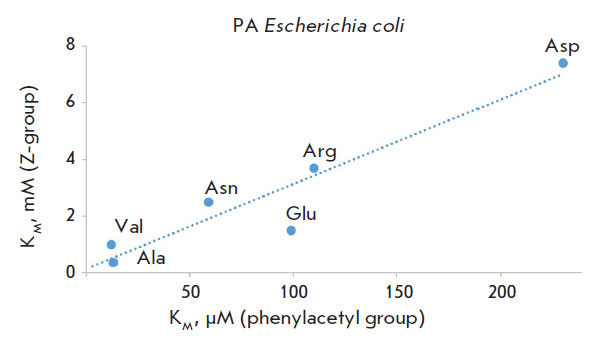
The correlation between the Michaelis constant (K_M_) values for the hydrolysis of the N-phenylacetyl (X axis) and N-benzyloxycarbonyl (Y axis) derivatives of amino acids catalyzed by penicillin acylase from *Eschericha coli *

**Fig. 3:**
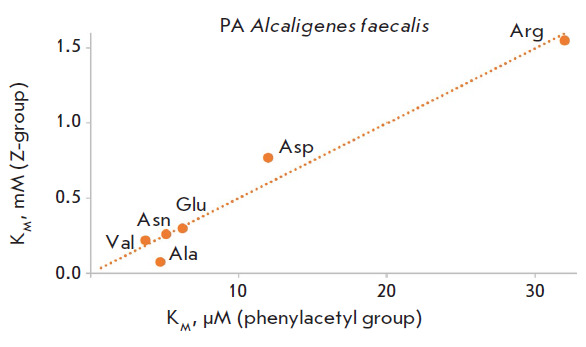
The correlation between the Michaelis constant (K_M_) values for the hydrolysis of the N-phenylacetyl (X axis) and N-benzyloxycarbonyl (Y axis) derivatives of amino acids catalyzed by penicillin acylase from *Alcaligenes faecalis *

The difference in acyl groups per oxygen atom significantly alters the efficiency of substrate binding in the active center of penicillin acylases, which is characteristic of both Alcaligenes faecalis penicillin acylase and Escherichia coli penicillin acylase. However, the change in the structure of the N-acyl group does not affect the enzymes’ specificity toward the amino acid side chain radical as evidenced by the correlations between the Michaelis constants for the reactions of removal of the N-phenylacetyl and N-benzyloxycarbonyl protecting groups catalyzed by both penicillin acylases. Meanwhile, both enzymes differ in their specificities towards this structural fragment, as shown in Fig. 2 and Fig. 3, where the least effective binding substrates for penicillin acylase from Escherichia coli are derivatives of aspartic acid, while for penicillin acylase from Alcaligenes faecalis they are arginine derivatives.

When the phenylacetyl residue is replaced with benzyloxycarbonyl, the affinity of both enzymes for the substrate decreases by more than an order of magnitude, while penicillin acylase from Alcaligenes faecalis exhibits a higher affinity for new substrates (the K_M_ values lie in the range of 0.08–1.6 mM).

**Fig. 4:**
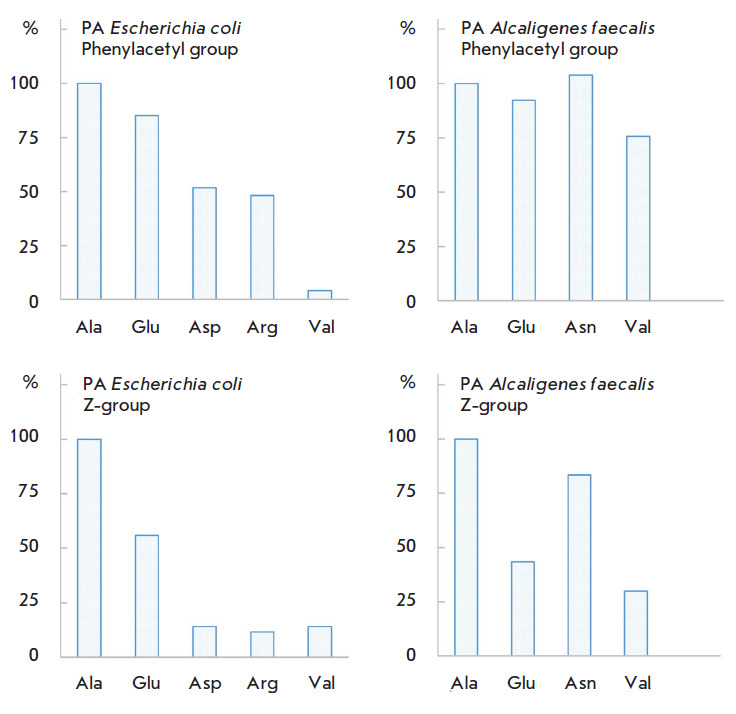
The catalytic activity of penicillin acylases from *Eschericha coli *(left-hand side figures) and *Alcaligenes faecalis *(right-hand side figures) in the hydrolysis of the N-phenylacetyl and N-benzyloxycarbonyl (Z) amino acid derivatives expressed as the catalytic constant value (%) with respect to the corresponding alanine derivatives: the upper and lower graphs demonstrate the activity of the penicillin acylases with respect to the N-phenylacetyl and N-benzyloxycarbonyl derivatives of amino acids, respectively

**Fig. 5:**
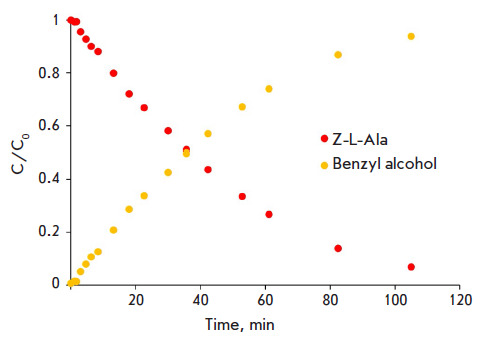
Removal of the N-benzyloxycarbonyl protecting group catalyzed by penicillin acylase from *Alcaligenes faecalis*. The red dots show the changes in the concentration of the substrate (N-benzyloxycarbonyl-L-Ala); the yellow ones indicate the accumulation of the reaction product (benzyl alcohol). Reaction conditions: pH 7.5; 25°C; substrate concentration, 1 mM; enzyme concentration, 6 μM

Whereas the affinity of both enzymes for substrates depends on the nature of the amino acid side chain, this structural fragment affects the reactivity of penicillin acylases in different ways Fig. 4). The nature of the amino acid side chain in the series of N-phenylacetyl derivatives of amino acids has little effect on the catalytic activity of penicillin acylase from Alcaligenes faecalis (the upper right-hand side of the figure), while the reactivity of penicillin acylase from Escherichia coli strongly depends on this structural fragment and drops more than 20-fold in the series N-Phac-Ala, Glu, Asp, Arg, and Val (the upper left-hand side of the figure). When the phenylacetyl residue is replaced with benzyloxycarbonyl, the reactivity of the enzymes decreases. Thus, the activity of penicillin acylase from Escherichia coli decreases 10- to 49-fold depending on the structure of the side chain radical of the amino acid residue while penicillin acylase from Alcaligenes faecalis is even more sensitive to this structural change: the enzyme activity decreases by two orders of magnitude. Nevertheless, both penicillin acylases are able to remove the N-benzyloxycarbonyl protecting groups in the amino acid derivatives (Fig. 5); by introducing mutations in the enzyme structure, one can enhance the catalytic activity to these nonspecific substrates. The experience in studying penicillin acylase from Escherichia coli shows that both the catalytic activity and the affinity for the substrates can be improved by protein engineering [22].

## CONCLUSIONS

This study demonstrated that changes in the structure of the N-acyl group in N-acylated amino acid derivatives have a significant impact on both the recognition and activity of penicillin acylases with respect to this series of substrates. Nevertheless, both enzymes (namely, penicillin acylase from Alcaligenes faecalis and penicillin acylase from Escherichia coli) can efficiently remove the N-benzyloxycarbonyl protecting group in amino acid derivatives under mild conditions without the use of toxic reagents, which makes them useful in organic synthesis. The efficiency of such biocatalytic deprotection can be further enhanced using modern methods of rational enzyme design.

## Abbreviations

PA: penicillin acylase
HPLC: high-performance liquid chromatography
PMSF: phenylmethylsulfonyl fluoride
NIPAB: 2-nitro-5-[(phenylacetyl)amino]benzoic acid
o-FA: o-phtalaldehyde
NAC: N-acetyl-L-cysteine
Z: benzyloxycarbonyl protective group
